# Case series: Joubert syndrome and eosinophilic esophagitis

**DOI:** 10.1002/jpr3.70132

**Published:** 2026-01-05

**Authors:** Jonathon Schening, Stephanie Leon‐Paredes, Eric Chiou, Vibha Szafron, Anthony Olive, Sara Anvari

**Affiliations:** ^1^ Division of Allergy, Immunology, and Retrovirology Texas Children′s Hospital Houston Texas USA; ^2^ Department of Pediatrics Baylor College of Medicine Houston Texas USA; ^3^ Division of Gastroenterology and Nutrition Texas Children′s Hospital Houston Texas USA

**Keywords:** ciliopathy, dysmotility, eosinophilic gastrointestinal diseases (EGID), genetic disease

## Abstract

Joubert syndrome (JS) is a rare genetic disorder characterized by developmental abnormalities, particularly in the brainstem and cerebellar vermis, alongside multisystem manifestations such as kidney and liver anomalies, polydactyly, cleft lip or palate, and tongue defects. The underlying ciliopathy causing JS may also contribute to gastrointestinal symptoms and immune dysregulation via Wnt signaling and impaired epithelial maintenance. Dysmotility including Hirschsprung disease has been documented at increased rates in JS and in other ciliopathies. Our case series highlights how JS patients frequently exhibit feeding intolerance, vomiting, and poor growth, which may raise suspicion for an underlying gastrointestinal condition, such as eosinophilic esophagitis (EoE). Gastrointestinal symptoms often overlap with other chronic issues, delaying diagnosis and treatment, which can affect long‐term outcomes. These cases underscore the importance of thorough evaluations, including endoscopy, to investigate persistent symptoms suggestive of eosinophilic gastrointestinal diseases (EGIDs)/EoE. Such vigilance promotes early targeted therapies, improves quality of life, and decreases the risk of complications including formation of esophageal strictures.

## Abstract (highlights)

**What is Known:**

- Joubert syndrome (JS) is a rare, heterogeneous genetic ciliopathy which has been previously associated with dysmotility including Hirschsprung disease.
- Diagnosis of eosinophilic esophagitis (EoE) requires a high index of suspicion and requires endoscopy and adequate biopsy samples.

**What is New:**

- JS may have gastrointestinal complications beyond dysmotility and Hirschsprung disease.
- There may be a link between JS and EoE which would benefit from early endoscopy, diagnosis, and treatment.
- The Wnt signaling pathway is important to epithelial maintenance and is impaired in ciliopathies; this may be a causal link between JS and EoE.

## 1 INTRODUCTION

Joubert syndrome (JS) is a rare autosomal recessive genetic ciliopathy estimated to affect fewer than 5000 patients in the United States.^1^ Clinical characteristics include abnormal brain development, especially brainstem and cerebellar vermis; kidney and liver anomalies including progressive fibrosis; physical defects including polydactyly, cleft lip or palate, and tongue anomalies. More than 35 discrete genes have been associated with JS, and many patients remain without a genetic diagnosis; the myriad of culprit genes likely contributes to the phenotypic heterogeneity of JS.^2^, ^3^, ^4^, ^5^ Though dysmotility including Hirschsprung disease has been documented, failure to thrive and eosinophilic gastrointestinal diseases (EGID), particularly eosinophilic esophagitis (EoE), have not been reported in the literature to our knowledge.^2^ We present a case series of two patients with JS with failure to thrive and feeding difficulties who were diagnosed with EoE.

## 2 CASE 1

A 4‐year‐old boy with JS (*OFD1*: Xp22.2 partial deletion), allergic rhinitis, asthma, and primary ciliary dyskinesia, initially presented with symptoms of persistent drooling, chronic wet cough, frequent upper and lower respiratory tract infections, and poor feeding progression. For presumed gastroesophageal reflux disease (GERD), he was started on Lansoprazole 15 mg once daily. He subsequently underwent a triple endoscopy while on lansoprazole, including esophagogastroduodenoscopy (EGD), bronchoscopy, and rhinoscopy at 4 years of age for continued symptoms despite proton pump inhibitor (PPI) therapy. His EGD findings demonstrated middle‐distal esophageal furrowing and edema. Distal esophageal biopsy demonstrated a peak of 22 eosinophils per high‐power field (eos/hpf), and mid‐esophageal biopsy demonstrated rare intraepithelial eosinophils; proximal esophageal biopsy was not performed at this time. His PPI regimen was increased to high‐dose, with esomeprazole 20 mg twice daily for 3 months without symptomatic change. He was weaned off PPI and started on oral viscous budesonide (OVB) 0.5 mg twice daily. While symptoms of drooling and throat clearing partially improved, he continued to have feeding difficulty and was unable to advance his diet past puree consistency. A repeat EGD performed 11 months after starting OVB demonstrated patchy erythema, ulcerations, friability, and a peak of 2 eos/hpf in the distal esophagus. Endoscopy and histology were normal in the mid and proximal esophageal, antral, and duodenal mucosa. He was continued on OVB 0.5 mg twice daily; famotidine 20 mg daily was added for his continued cough and possible GERD. Two months later, famotidine was transitioned to lansoprazole 20 mg twice daily, which did not help the persistent coughing symptoms that occurred after his feeds. Multiple videofluoroscopic swallow studies did not reveal penetration or aspiration.

Most recently, he continues with a pureed diet and has coughing with feeds. He was started on bethanechol 4 mg three times daily before meals for motility, lansoprazole was reduced back to 20 mg once daily, and OVB was continued at 0.5 mg twice daily. He remains on these medications, with scheduled follow‐up with Allergy and Immunology, Gastroenterology, Pulmonology, and ENT; he awaits scheduling for follow‐up endoscopy.

## 3 CASE 2

A 4‐year‐old boy with JS (biallelic *CEP41* variants), short stature (receiving growth hormone), asthma, and eczema presented for multiple IgE‐mediated food allergies, gastrostomy tube‐dependence for feeding intolerance, vomiting, and poor growth. At 15 months of age, the patient was started on esomeprazole 5 mg twice daily due to suspected gastroesophageal reflux. Parents stopped esomeprazole as there was some improvement in the vomiting episodes, but he continued to have a persistent cough. Due to his persistent symptoms, he was evaluated in a multidisciplinary Aerodigestive program (includes specialists in GI, ENT, and Pulmonary). A videofluoroscopic swallow study revealed silent aspiration, nasopharyngeal reflux, and a cricopharyngeal bar.

Following these findings, the patient underwent a triple scope including EGD which showed erythema in the distal esophagus and diffuse gastropathy. Histological evaluation was notable for active esophagitis (32 eos/hpf in the mid esophagus and 5 eos/hpf in the distal esophagus), raising concern for EoE. The patient was restarted on esomeprazole 10 mg twice daily and referred to a multidisciplinary EGID clinic with gastroenterology, allergy & immunology and dietician given his EoE diagnosis and IgE‐mediated food allergies to eggs and peanuts. The patient demonstrated some improvement on high‐dose PPI but continued to have intermittent choking episodes for which he underwent dairy elimination. On dairy elimination, vomiting stopped, and repeat EGD 7 months later was grossly and histologically normal. He has shown improvement in his weight gain and growth. He continues feeding and occupational therapy. Overall, EGID symptoms have remained under control with 3 food elimination diet (eggs, peanuts, and dairy).

## 4 DISCUSSION

JS is a rare, genetically heterogeneous ciliopathy with multisystem involvement, including characteristic brain malformations, kidney and liver anomalies, physical defects, feeding difficulties thought to be secondary to bulbar dysfunction and gastrointestinal complications. While gastrointestinal dysmotility is a recognized manifestation, the association between JS and eosinophilic gastrointestinal EGIDs, particularly EoE, remains underexplored. While EoE is estimated to affect approximately 1 patient per 1000–2000 in the general population, these two cases reflect 2 out of 84 patients diagnosed with JS at our tertiary pediatric center. Notably, the majority of these patients have not undergone diagnostic EGDs. The heterogeneous genes causing JS (X‐linked in case 1, autosomal recessive on chromosome 7 in case 2) may overlap atopy‐related loci, including those influencing EoE.

Ciliary structures are integral to Wnt pathway modulation, and mutations affecting ciliary function could disrupt this signaling, leading to impaired epithelial maintenance and repair.^6^ Given the known role of cilia in cellular signaling and epithelial homeostasis, it is plausible that patients with ciliopathies such as JS may have intrinsic defects in epithelial barrier function that predispose them to EGID. The Wnt signaling pathway, which regulates epithelial self‐renewal and differentiation, has been shown to be dysregulated in EoE, with downregulation evidenced by increased expression of the Wnt inhibitor SFRP1 and decreased downstream targets such as Cyclin D1 and c‐Myc. Disruptions in this pathway could contribute to impaired epithelial repair, increased antigen exposure, and chronic inflammation, further linking ciliary dysfunction to EoE pathogenesis. ^5^

These cases illustrate the spectrum of gastrointestinal and feeding challenges in JS. Both patients presented with feeding difficulties, poor growth, and chronic cough, initially attributed to gastroesophageal reflux or decreased oromotor skills with concern for aspiration. However, endoscopic evaluations revealed histologic evidence of EoE, underscoring the importance of thorough investigations, including EGD, in this population. Moreover, consistent biopsies of proximal, mid, and distal esophageal sites in patients with any index of suspicion for EoE will likely increase diagnostic yield and mitigate uncertainty portended by coincidence of eosinophilic infiltration of the distal esophagus in both EoE and GERD.

## 5 CONCLUSION

There is no definitive data to indicate JS causes EoE or other EGIDs, and However, it remains important to consider noneosinophilic culprits: Case 1 illustrates that despite treatment for EoE, dysphagia and postprandial cough may persist and may be mediated by esophageal dysmotility or a neurogenic dysphagia, common in JS. Comorbid EoE may go unnoticed, resulting in delayed or suboptimal treatment and complications including fibrostenotic disease or chronic feeding problems despite histologic remission of disease. Recognizing and addressing EGIDs in this population can enable prompt diagnosis with targeted therapies, prevent long‐term complications, and lead to an improvement in quality of life.

## CONFLICT OF INTEREST STATEMENT

Vibha Szafron received research grant funding from FARE and the AAP for work with the Houston Independent School District (HISD). Sara Anvari receives research grant funding from NIH/NIAID, Regeneron, DBV Technologies, and Astra Zeneca. Sara Anvari also serves on the medical advisory board for the International FPIES Association (iFPIES).

## ETHICS STATEMENT

Informed patient consent was obtained for both cases.
